# Association Between Emphysema and Breast Cancer: Data from National Health and Nutrition Examination Survey (1998–2016)

**DOI:** 10.1177/26884844251359511

**Published:** 2025-07-15

**Authors:** Li Jianglong, Xiao Jiabiao, Liu Zhentian, Wang Yaqi, Yan Wei, Chen Tanxiu

**Affiliations:** ^1^ Department of breast surgery, Jiangxi Cancer Hospital and Institute, Jiangxi Clinical Research Center for Cancer, The Second Affiliated Hospital of Nanchang Medical College, Jiangxi Key Laboratory of Oncology, Nanchang, Jiangxi, China.; ^2^ Department of Hematology Oncology, Taihe County People’s Hospital, Taihe, Jian, Jiangxi, China.; ^3^ Department of Thoracic Oncology, Jiangxi Cancer Hospital and Institute, Jiangxi Clinical Research Center for Cancer, The Second Affiliated Hospital of Nanchang Medical College, Jiangxi Key Laboratory of Oncology, Nanchang, Jiangxi, China.; ^4^ Department of General Oncology, Jiangxi Cancer Hospital and Institute, Jiangxi Clinical Research Center for Cancer, The Second Affiliated Hospital of Nanchang Medical College, Jiangxi Key Laboratory of Oncology, Nanchang, Jiangxi, China.; ^5^ Jiangxi Academy of Clinical Medical Sciences, Institute of Neurology and Department of Neurology, Jiangxi Key Laboratory of Neurological Diseases, The First Affiliated Hospital, Jiangxi Medical College, Nanchang University, Nanchang, Jiangxi, China.

**Keywords:** breast cancer, emphysema, NHANES, risk stratification analysis

## Abstract

**Background:**

Emphysema has been linked to an elevated risk of lung cancer, yet the association between emphysema and breast cancer (BC) has not been established. Hence, our study aimed to investigate the potential correlation between emphysema as an exposure factor and BC as the outcome, while accounting for various covariates associated with BC.

**Methods:**

Participants from the National Health and Nutrition Examination Survey database from 1998 to 2016 were selected for analysis. Initially, ineligible individuals were excluded from this analysis. Subsequently, chi-square test and *t*-test were independently executed to assess variances in covariates and exposure factors between patients with BC and controls, leading to the development of a baseline table. Subsequently, weighted multivariate logistic regression analysis was conducted to explore the potential association between emphysema and BC, resulting in the development of three logistic regression models. Additionally, risk stratification analysis using weighted stratified logistic regression was performed to analyze the impact of emphysema on BC across different populations.

**Results:**

After excluding ineligible individuals, 4,937 participants remained, comprising 152 patients with BC and 4,785 controls. The baseline table revealed significant differences between BC and control samples in terms of emphysema (*p* = 1 × 10^−2^), age (*p* = 5 × 10^−4^), race (*p* = 1 × 10^−3^), marital status (*p* = 5 × 10^−4^), hypertension (*p* = 5 × 10^−4^), and number of pregnancies (*p* = 2.1 × 10^−2^). Furthermore, all *p* values for emphysema across the three model types were less than 0.05, indicating that the association between emphysema and BC was not significantly affected by other covariates. Meanwhile, the risk stratification analysis demonstrated that emphysema may be a risk factor for BC (odds ratio = 2.6, 95% confidence interval 1.25–5.41, *p* = 1.09 × 10^−2^).

**Conclusion:**

The study’s findings indicating a correlation between emphysema and BC, with emphysema may be a risk factor for BC. This provides a potential theoretical basis for the development of BC treatment strategies.

## Introduction

Breast cancer (BC), the leading cause of cancer-related mortality among women globally, remains a serious threat to human health. According to data from the World Health Organization, over 2.3 million new cases and 670,000 death from BC were reported in 2020, accounting for 11.7% of all cancer cases.^1^ In both developing and developed countries, the incidence and mortality rates of BC continue to rise.^2^ Numerous factors have been confirmed to be associated with the development or progression of BC, according to epidemiological research.^3^ The primary factors contributing to high incidence and mortality from BC in underdeveloped countries include inadequate medical facilities, delayed diagnosis, insufficient screening programs, and a lack of awareness or knowledge.^4^ Developing effective treatment options in clinical practice requires a thorough understanding of the factors linked to the advancement of BC in order to mitigate its significant impact on global health.^3^ Providing BC patients with a clear diagnosis and prognosis is vital for accurately educating them about the course of the disease and placing them in appropriate treatment.

Emphysema is a component of chronic obstructive pulmonary disease (COPD) that severely reduces quality of life. It is a potentially fatal condition characterized by airflow restriction and lung inflammation, primarily due to damage to the alveolar walls and small airways.^5^ Recent retrospective cohort studies have demonstrated that patients with emphysema have an increased risk of lung cancer.^6^ Additionally, having emphysema or COPD is a strong indicator of poor prognosis for lung cancer patients.^7^ Early diagnosis of these diseases should be considered in relation to lung cancer.^8^ Moreover, HIV patients with emphysema have been found to have a higher mortality rate.^9^ However, the impacts of emphysema on BC prognosis remain unclear. One case report suggests that fascial blocks might be the best strategy for BC patients with bullous emphysema, indicating that emphysema may increase the surgical risks for these patients.^10^ Furthermore, it has been reported that patients receiving A1PI therapy for α1-antitrypsin-deficient emphysema experienced one death (from respiratory failure), while those receiving placebo therapy had three deaths (from sepsis, pneumonia, and metastatic BC).^11^ The study have also indicated that women with relatively low respiratory quotient have a significantly increased risk of BC after menopause.^12^ To the best of our knowledge, there are no studies that explore the potential association between emphysema and BC. Therefore, the relationship between emphysema and BC needs to be further investigated.

To bridge this knowledge gap, we utilized the extensive National Health and Nutrition Examination Survey (NHANES) dataset to assess the relationship between emphysema and BC in this study. A baseline table was created, and the differences in clinical features between the control group and the BC group were analyzed using chi-square tests and *t*-tests. Additionally, we established three multivariate regression models and conducted risk stratification analysis. The results of the present study may provide a theoretical basis for the treatment and prevention of BC, ultimately improving public health.

## Materials and Methods

### Data extraction

NHANES is an annual study conducted in the United States of America. It purposes to estimate the health and nutritional status of adults by collecting data and information related to health. All NHANES participants must provide written consent before participating in the survey, and all data are available online.

The inclusion criteria for this analysis involved 92,062 participants from the period of 1998 to 2016. Initially, the data were cleaned according to covariates such as sex, age, marital status, education, and alcohol consumption to exclude data that lacked clinical information. The data cleaning process was shown in Supplementary Table S1. Then, certain participants were excluded based on specific conditions: those under 18 years of age, males, individuals with gynecological, endocrine disorders, or other types of cancers, subjects who received antibiotics or any neoadjuvant therapy within three months. The remaining participants were included for subsequent analysis.

### Definition of variables

In this study, emphysema was considered as the exposure factor, and BC was the outcome. BC was characterized by participants being queried with the question, “Has a doctor or any other health professional ever informed you that you have cancer or any type of malignancy?” Those who responded affirmatively were subsequently asked, “What kind of cancer is this?” Only respondents who reported BC as their primary and single tumors were included. In a similar manner, emphysema was identified as individuals who responded positively to the query “Has a doctor or other health professional ever informed you that you have emphysema?”

Additionally, several covariates were employed in this study. Among them, household income poverty ratio and number of pregnancies were continuous variables without grouping criteria. Meanwhile, some classified variables were employed. Specifically, age included ≤50 and >50 groups. Race encompassed Mexican American, Non-Hispanic Black, Non-Hispanic White, other Hispanic, and other races (including multiracial). Marital status contained never married, married, and divorced. The education level encompassed the following categories: less than 9th grade, 9–11th grades (including 12th grade with no diploma), high school graduate/GED or equivalent, some college or associate degree (AA degree), and college graduate or above. Body mass index (BMI = weight/height^2^) included normal (18.5–30.0 kg/m^2^), underweight (<18.5 kg/m^2^), and obesity (>30 kg/m^2^). Hypertension was categorized as yes and no. Alcohol drinks within the past 12 months was also divided into yes and no.

### Statistical analysis

To explore the difference in the included variables and exposure factors between patients with BC and controls, chi-square test and *t-test* were employed to analyze and construct a baseline table by “tableone” (v 0.13.2)^13^ (*p* < 0.05).

Following this, to examine the relationship between emphysema and BC, weighted multivariate logistic regression analysis was applied to construct three logistic regression models by “nhanesA” (v 1.0)^14^ (*p* < 0.05). Specifically, Model 1 solely accounted for emphysema without adjusting for any related covariates. In Model 2, adjustments were made for age and race. Subsequently, Model 3 incorporated BMI, marital status, education level, hypertension, alcohol drinks, household income poverty ratio, and number of pregnancies into Model 2. Notably, odds ratio (OR) and 95% confidence interval (CI) were essential parameters for determining the relationship.

Furthermore, to analyze the effects of emphysema on BC within various crowd, weighted stratified logistic regression was employed to conduct risk stratification analysis by “survey” (v 4.2.1)^15^ (*p* < 0.05).

In general, all analyses were clearly executed *via* R software (v 4.2.2). Two-tailed tests were clearly employed with a notably level set at *p* < 0.05.

## Results

### Characteristics of participants

**FIG. 1.**
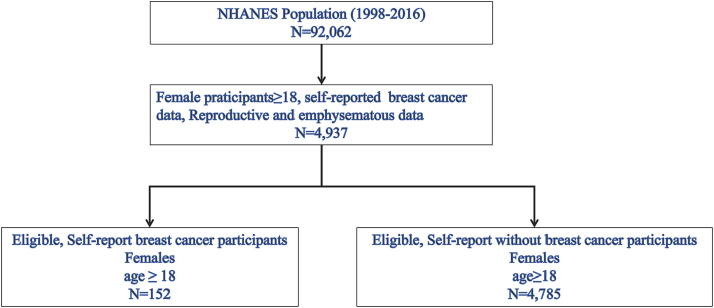
Flowchart of the sample selection from the NHANES (1998–2016). NHANES, National Health and Nutrition Examination Survey.

Among the initial 92,062 participants, a total of 4,937 individuals met the inclusion criteria after applying the exclusion criteria outlined in Figure 1. This group included 152 self-reported BC participants and 4,785 self-reported participants without BC. To clarify the clinical characteristics of these individuals, the baseline characteristics of the two groups were analyzed by weighted chi-square tests or weighted t-tests. Table 1 and 2 indicate the clinical characteristics of the participants with and without BC. The BC and without BC groups were compared by weighted chi-square test or weighted *t-test* and significant differences were found between variables, including emphysema (*p* = 0.01), age (*p* < 0.001), race (*p* = 0.001), marital status (*p* < 0.001), hypertension (*p* < 0.001), and number of pregnancies (*p* < 0.05). Participants with BC were more frequently ≥50 years (94.1%), non-Hispanic white (50.0%), divorced (44.1%), hypertension (68.4%), and emphysema (3.9%), and less frequently pregnancies (1.7%).

**Table 1.** Characteristics of Participants from the NHANES (1998–2016)

	Characteristics	State	Normal	Breast cancer	Normal rate	Breast cancer rate	*p* value
	Age	≤50	2126 (44.4)	9 (5.9)	44.4	5.9	<0.001
	Age	>50	2659 (55.6)	143 (94.1)	55.6	94.1	<0.001
	Race	Mexican American	1060 (22.2)	16 (10.5)	22.2	10.5	0.001
	Race	Other Hispanic	437 (9.1)	11 (7.2)	9.1	7.2	0.001
	Race	Non-Hispanic White	1597 (33.4)	76 (50)	33.4	50	0.001
	Race	Non-Hispanic Black	1182 (24.7)	33 (21.7)	24.7	21.7	0.001
	Race	Other race, including multi-racial	509 (10.6)	16 (10.5)	10.6	10.5	0.001
	Marital_Status	Never married	494 (10.3)	5 (3.3)	10.3	3.3	<0.001
	Marital_Status	Married	2976 (62.2)	80 (52.6)	62.2	52.6	<0.001
	Marital_Status	Divorced	1315 (27.5)	67 (44.1)	27.5	44.1	<0.001
	Education_Level	Less Than 9th Grade	822 (17.2)	22 (14.5)	17.2	14.5	0.4
	Education_Level	9–11th Grade (Includes 12th grade with no diploma)	810 (16.9)	22 (14.5)	16.9	14.5	0.4
	Education_Level	High School Grad/GED or Equivalent	1114 (23.3)	31 (20.4)	23.3	20.4	0.4
	Education_Level	Some College or AA degree	1261 (26.4)	48 (31.6)	26.4	31.6	0.4
	Education_Level	College Graduate or above	778 (16.3)	29 (19.1)	16.3	19.1	0.4
	BMI	Normal	2640 (55.2)	90 (59.2)	55.2	59.2	0.6
	BMI	Underweight	63 (1.3)	2 (1.3)	1.3	1.3	0.6
	BMI	Obesity	2082 (43.5)	60 (39.5)	43.5	39.5	0.6
	hypertension	No	2768 (57.8)	48 (31.6)	57.8	31.6	<0.001
	hypertension	Yes	2017 (42.2)	104 (68.4)	42.2	68.4	<0.001
	alcohol_drinks	No	2441 (51)	83 (54.6)	51	54.6	0.4
	alcohol_drinks	Yes	2344 (49)	69 (45.4)	49	45.4	0.4
	Breast cancer	NO	4785 (100)	0 (0)	100	0	<0.001
	Breast cancer	Yes	0 (0)	152 (100)	0	100	<0.001
	Emphysema	No	4731 (98.9)	146 (96.1)	98.9	96.1	0.01
	Emphysema	Yes	53 (1.1)	6 (3.9)	1.1	3.9	0.01

Categorical variables are displayed as numbers (percentages); Normally distributed continuous variables are presented as means ±  SD.

BMI, Body mass index; NHANES, National Health and Nutrition Examination Survey; SD, standard deviation.

**Table 2. Comparison of household income poverty ratio and number of pregnancies between breast cancer and control groups:** 

	Characteristics	Normal	Breast cancer	*p* value
1	Household income poverty ratio	2.2 (1.5)	2.4 (1.4)	0.23
2	Time of pregnant	3.7 (2.3)	3.3 (1.7)	<0.05

### Association between emphysema and BC

To further determine the relationship between exposure factors and BC, we used the R package “NHANES” to examine the association of these factors with BC. We then performed a weighted multivariate logistic regression analysis (Table 3). Our analysis revealed a positive association between emphysema and BC. All *p* values for emphysema in the three models were less than 0.05, indicating that the impact of emphysema on BC was not significantly affected by other covariates. Specifically, the results of model 1 were OR = 3.83, 95% CI = 1.82–8.03, *p* < 0.001, the results of model 2 were OR = 2.62, 95% CI = 1.25–5.51, *p* < 0.05, and the results of model 3 were OR = 2.60, 95% CI = 1.25–5.41, *p* < 0.05.

**Table 3.** Associations Between the Emphysema and BC Among Participants from the NHANES (1998–2016)

	Model 1	Model 2	Model 3
	OR (95% CI) *p* value	OR (95% CI) *p* value	OR (95% CI) *p* value
Emphysema			
	No	Reference	Reference	Reference
	Yes	3.83 (1.82,8.03)^***^	2.62 (1.25,5.51)^*^	2.60 (1.25,5.41)^*^

Data are presented as OR (95% CI).

Model 1 = No adjust.

Model 2 = Model 1 plus adjusted for age and race.

Model 3 = Model 2 plus adjusted for BMI, marital status, education level.

*p < 0.05, ***p < 0.001.

CI: confidence interval; OR: odds ratio.

**FIG. 2.**
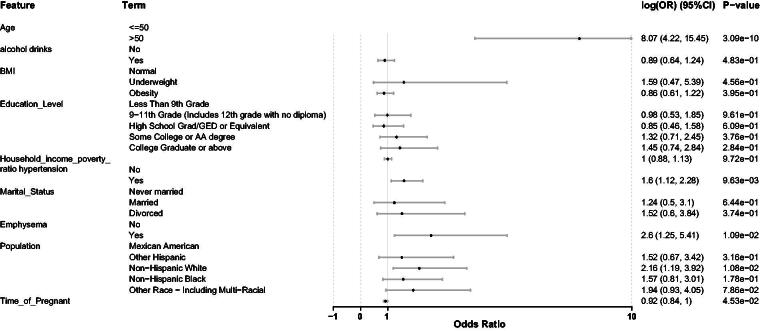
Baseline characteristics of the study population.

To further confirm the stability of the correlation between exposure factors and BC risk across different races. Based on the selected exposure factors, stratified analysis was conducted through weighted stratified logistic regression to further evaluate the association between exposure factors and BC. Additionally, the combined risk stratification analysis produced a forest plot that similarly demonstrated that emphysema increased BC risk (OR = 2.6, 95% CI: 1.25–5.41, *p* < 0.05) (Fig. 2).

To exclude the influence of smoking on the results, we conducted a comprehensive review of relevant literature and datasets to collect detailed smoking history information, including current smokers, former smokers, and never smokers. However, after incorporating smoking as a covariate in our survival analysis, we found that the *p* value for the association between smoking status and BC was 0.43, substantially higher than 0.05 (Supplementary Fig. S1). This indicates no significant correlation between smoking and BC outcomes in our cohort. Furthermore, our in-depth literature review revealed that smoking status was not significantly associated with overall BC risk in multivariate-adjusted models.^16^

Regarding emphysema duration, the only available relevant information in the database was the age at which patients were diagnosed with emphysema. We attempted to incorporate this variable into our analysis. However, this reduced our sample size to just 72 cases. Among these 72 samples, one case had missing data, and of the remaining 71 samples, only 8 cases were positive for the outcome while 63 were negative (Supplementary Table S2). According to statistical principles, such a small sample size is far from sufficient to conduct reliable and effective analyses. Small sample studies often come with high sampling errors and low statistical power, which could lead to inaccurate and non-generalizable results. Therefore, based on the current data, we cannot further investigate the relationship between emphysema duration and BC.

As shown in the Supplementary Tables S3 and Supplementary Table S4, there is a significant association between COPD and emphysema, both being pulmonary diseases. However, no significant associations were found between emphysema and the remaining diseases. We included diseases and cancers with relatively large sample sizes in our analysis to ensure reliability and statistical power. While COPD showed a significant association with emphysema, no significant associations were found between emphysema and other diseases in our study.

Overall, we found a significant association between emphysema and BC, with emphysema may be a risk factor for BC. This finding may be helpful in BC treatment.

## Discussion

In this study, the results of multiple regression analysis revealed a correlation between emphysema and BC. By examining the baseline statistical table, we discovered significantly differences in the emphysema population sizes between patients with BC and control groups, indicating that emphysema affects BC significantly. Furthermore, there were notable variations in the effect of emphysema on BC across the three model modifications, suggesting that other factors had no discernible effect on the influence of emphysema on BC. Using risk stratification analysis, we discovered that emphysema has an OR value greater than 1, indicating that it is a risk factor for BC.

Several studies have reported the association between tumors and respiratory diseases. A previous study found that cancer patients with SARS-CoV-2 Omicron infections typically exhibit mild clinical symptoms. However, in certain cases, SARS-CoV-2 Omicron infections can lead to severe illness or even death; therefore, early infection management and vigilant observation are important.^17^ SARS-CoV-2 infection adversely affects the prognosis of pan-tumor cases by impacting immunity.^18^ Currently, BC is the most prevalent malignant tumor among women.^19^ In addition, there are several risk factors that affect the prognosis of BC.^20^ Oluwatosin A Ayeni et al. reported that South African women who are diagnosed with BC often have other chronic diseases. Compared to nonobese women, obese women had a higher overall survival rate. However, women with obesity, diabetes, heart disease, and HIV had shorter survival times than those without these conditions.^21^ It was also reported that underwent autologous (ABR) and implant-based breast reconstruction (IBR) poor outcomes are linked to COPD, an independent risk factor. Specifically, patients with COPD undergoing ABR were more likely to experience respiratory problems and incurred higher overall hospital costs. Meanwhile, there was an increased risk of deep wound problems, renal issues, and out-of-hospital transfers among COPD patients receiving IBR.^22^ In a study, Chien et al. found that a 53-year-old woman experienced respiratory distress with increasing desaturation and hemoptysis following axillary lymph node dissection and a left simple mastectomy for left BC.^23^ These findings suggested that respiratory diseases may be a risk factor for BC.

Pulmonary emphysema is a chronic lung disease characterized by persistent abnormal air content, excessive swelling, and damage to the airway wall. The primary cause of emphysema is recognized to be cigarette smoking.^24^ For middle-aged and elderly patients with BC, current smokers had a lower survival rate than never smokers.^25^ A study showed that an increased risk of BC in women exposed to second-hand smoke.^23^ Two studies have provided evidence of the potential association between emphysema and BC. A study conducted by Sung Hwan Park et al. reported that a case of spontaneous pneumomediastinum and subcutaneous emphysema in patients receiving adjuvant treatment for BC that also included cryptogenic organizing pneumonia.^26^ In a clinical randomized controlled trial, Kenneth R Chapman founded that three deaths occurred in the placebo group (sepsis, pneumonia, and metastatic BC), while one occurred in the A1PI group (respiratory failure) among α1 antitrypsin deficiency emphysema patients.^11^ Our findings were consistent with previous studies. Using the NHANES database, we were the first to study the relationship between emphysema and BC among women. Pulmonary emphysema, primarily caused by smoking, shows significant associations with breast cancer (BC). Clinical evidence links emphysema to poorer BC outcomes, with our NHANES study being the first to establish this relationship in women, confirming previous findings.

Compared to previous studies, our study had several advantages. Our study explored the potential association between emphysema and BC, utilizing the NHANES database from 1998 to 2016, which included 4,937 participants. Through weighted multivariate logistic regression analysis, our research confirmed a correlation between emphysema as an exposure factor and BC as an outcome. The study considered multiple BC-related covariates and conducted risk stratification analysis to further examine how emphysema impacts BC incidence across different population groups. These findings provide a potential theoretical basis for developing BC treatment strategies.

This study has confirmed emphysema as a significant risk factor for BC. Given this finding, exploring novel therapies for patients with both conditions is of great importance. From a pathological mechanism perspective, both conditions exhibit dysregulated immune microenvironments, providing a theoretical basis for immunotherapy. Previous studies suggest that immunotherapeutic interventions could simultaneously enhance immune responses to combat cancer while improving respiratory function.^27,28^ Additionally, targeted therapy represents another promising novel treatment strategy.^29^ It was reported that certain growth factor-related signaling pathways, such as the epidermal growth factor receptor (EGFR) pathway, are abnormally activated in both emphysema and BC development. In emphysema, the abnormal activation of the pathway contributes to airway remodeling and alveolar destruction; in BC, it promotes tumor cell proliferation, migration, and invasion. Based on the association between emphysema and BC identified in this study, targeted therapeutic drugs addressing these common molecular pathways may become effective treatment options. Using small molecule inhibitors to block the EGFR pathway could potentially inhibit BC cell growth while simultaneously reducing emphysema-related lung lesion progression, providing patients with more precise and effective treatment options. Traditional chemotherapy and radiotherapy show clear limitations in BC patients with concurrent emphysema, as they can worsen lung function and increase the risk of complications.^30^ In contrast, novel therapies demonstrate distinct advantages: Immunotherapy offers high specificity and low toxicity while modulating immunity,^31^ and targeted therapy precisely inhibits tumors while minimizing interference with normal cells and protecting lung function.^32^ In conclusion, based on the close association between emphysema and BC identified in this study, novel therapeutic strategies such as immunotherapy and targeted therapy offer new treatment directions for this complex patient population. These therapies, by targeting common pathological mechanisms of both diseases, show promise for achieving more effective and safer treatment outcomes. However, the application of these novel therapies in patients with concurrent emphysema and BC remains in the exploratory phase. Future research needs to further clarify optimal treatment protocols, drug dosages, and combination therapy strategies to optimize therapeutic outcomes and improve patient survival rates and quality of life. Additionally, in-depth research into the common pathogenic mechanisms of both diseases will provide theoretical support for developing more innovative treatment approaches.

However, our study has several limitations. First, while using the NHANES database to analyze the association between emphysema and BC, we recognize potential selection bias due to sample selection and recruitment methods, and data reliance on self-reporting, especially with missing sensitive information like smoking history. Despite smoking being a crucial factor affecting emphysema and BC,^33,34^ we could not include it as a covariate due to data gaps. Second, although male BC incidence is low, this doesn’t mean men are entirely exempt. The database contained very few male BC cases, which were eliminated during data cleaning due to insufficient numbers, potentially influencing results. Third, it is important to note that our analysis of emphysema in patients with BC was based on a relatively small number of cases (6 out of 152 NHANES participants with BC). This limited sample size may reduce the statistical power of our analysis and could affect the generalizability of the findings. Future studies with larger cohorts are needed to confirm the observed associations and to further explore the potential link between emphysema and BC. Fourth, the frequent medical follow-ups and specialized monitoring for pulmonary and cardiac function in patients with BC, especially those who have received chest radiation or specific chemotherapies, could result in more detailed evaluations of comorbidities. This might influence the observed prevalence or severity of certain medical conditions in our study population. This highlights the need for future studies to account for such surveillance biases when comparing medical outcomes across different patient groups. Fifth, future studies should explore alternative methods for managing missing data, such as multiple imputation, to optimize the utilization of available data and reduce potential biases. Sixth, we used multivariate logistic regression to control for covariates and sample weights, minimizing potential bias and revealing a significant association between emphysema and BC. However, this method cannot directly prove causality. Future research should employ more rigorous methods for verification. Moreover, considering unmeasured confounding factors like smoking, future studies should incorporate more comprehensive covariate information, address sample selection bias, and enhance research accuracy and reliability. Ultimately, clinical trials will further confirm these findings and underlying mechanisms.

To summarize, this study combines the NHANES database to explore the relationship between emphysema and BC. The results showed significant differences in the impact of emphysema on BC under three model adjustments, indicating that the impact of emphysema on BC was not significantly affected by other covariates. The results of risk stratification analysis showed that the OR for emphysema was greater than 1, suggesting that emphysema may be a risk factor for BC.

## Conclusion

Patients with BC have a higher prevalence of emphysema compared with those without BC. Our findings highlight the importance of emphysema prevention and management in patients with BC.

## Data Availability

Publicly available datasets were analyzed in this study. The data can be found here: National Health and Nutrition Examination Survey (NHANES), https://wwwn.cdc.gov/nchs/nhanes/Default.aspx, NHANES, 1998–2016.

## Notes

### Abbreviations Used

A1PI: Alpha-1 Antitrypsin (α1-antitrypsin)
ABR: Autologous Breast Reconstruction
BC: Breast Cancer
BMI: Body Mass Index
CDC/NCHS: Centers for Disease Control and Prevention/National Center for Health Statistics
CI: Confidence Interval
COPD: Chronic Obstructive Pulmonary Disease
EGFR: Epidermal Growth Factor Receptor
GED: General Educational Development
IBR: Implant-Based Breast Reconstruction
NHANES: National Health and Nutrition Examination Survey
OR: Odds Ratio
SD: Standard Deviation
