# Supplementary figures and images for: Association Between Emphysema and Breast Cancer: Data from National Health and Nutrition Examination Survey (1998–2016)
Source: Womens Health Rep (New Rochelle). 2025 Jul 15;6(1):681–90. doi: 10.1177/26884844251359511 (PMC12479188; doi:10.1177/26884844251359511)

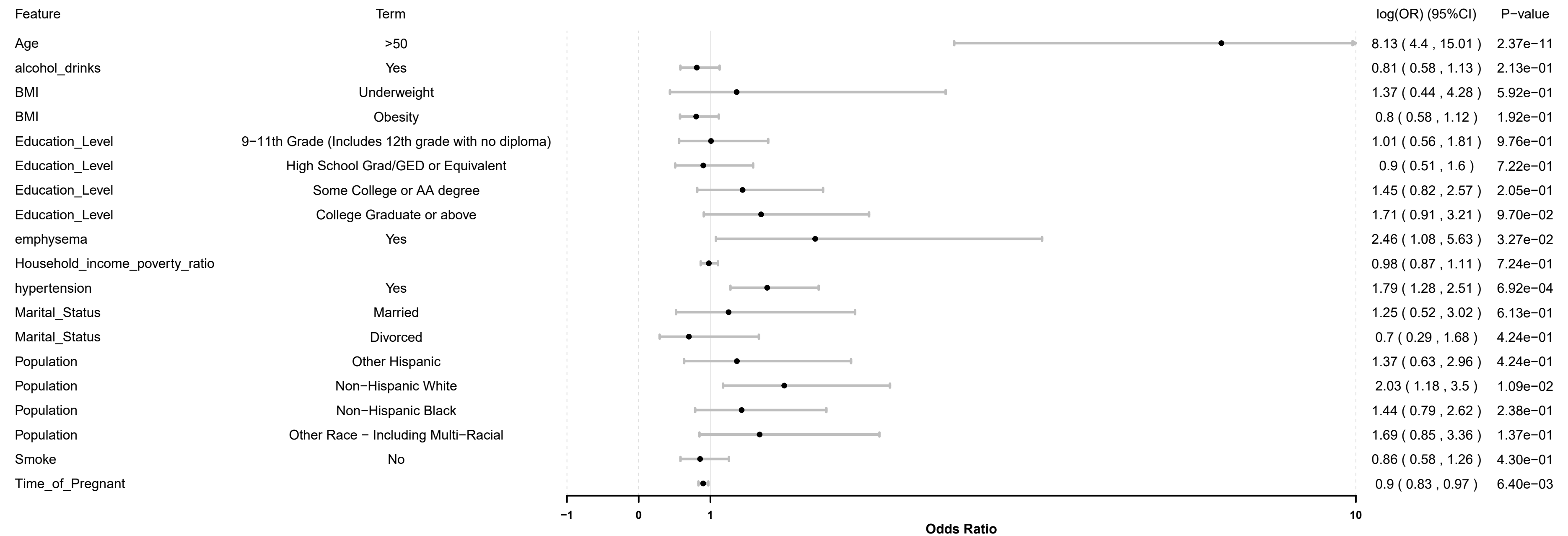

Supplement: Supplementary Figure S1 [file 26884844251359511_supplementary_figure_s1.pdf]
