# Supplementary material for: Association Between Emphysema and Breast Cancer: Data from National Health and Nutrition Examination Survey (1998–2016)
Source: Womens Health Rep (New Rochelle). 2025 Jul 15;6(1):681–90. doi: 10.1177/26884844251359511 (PMC12479188; doi:10.1177/26884844251359511)
Supplement: Supplementary Table S1 [file 26884844251359511_supplementary_table_s1.docx]

| **Covariant variable** | **Numbers** |
| --- | --- |
| All subjects from 1998 to 2016 | 92062 |
| sex | 39605 |
| Age | 39460 |
| Population | 39460 |
| Marital Status | 39460 |
| Education Level4937 | 20329 |
| BMI | 19056 |
| Hypertension | 14575 |
| Alcohol_drinks | 5729 |
| Emphysema | 4937 |

**Supplementary Table 1**
