# Supplementary material for: Association Between Emphysema and Breast Cancer: Data from National Health and Nutrition Examination Survey (1998–2016)
Source: Womens Health Rep (New Rochelle). 2025 Jul 15;6(1):681–90. doi: 10.1177/26884844251359511 (PMC12479188; doi:10.1177/26884844251359511)
Supplement: Supplementary Table S2 [file 26884844251359511_supplementary_table_s2.docx]

**Supplementary Table 2**

|  | **SEQN** | **Age** | **Population** | **Household income poverty ratio** | **Marital Status** | **Education Level** | **BMI** | **Number of Pregnancies** | **hypertension** | **alcohol drinks** | **breast_cancer** | **emphysema** | **emphysema_age** | **Smoke** |
| --- | --- | --- | --- | --- | --- | --- | --- | --- | --- | --- | --- | --- | --- | --- |
| 495 | 560 | >50 | Non-Hispanic White | 1.19 | Married | High School Grad/GED or Equivalent | Obesity | 2 | Yes | No | NO | 1 | 65 | Yes |
| 984 | 1090 | <=50 | Non-Hispanic White | 1.36 | Married | 9-11th Grade (Includes 12th grade with no diploma) | Underweight | 2 | No | No | NO | 1 | 44 | Yes |
| 2023 | 2247 | >50 | Non-Hispanic White | 2.26 | Married | High School Grad/GED or Equivalent | Normal | 3 | No | Yes | NO | 1 | 42 | Yes |
| 3376 | 3743 | >50 | Mexican American | 0.32 | Divorced | Less Than 9th Grade | Normal | 9 | Yes | Yes | NO | 1 | 76 | Yes |
| 4839 | 5341 | >50 | Non-Hispanic White | 0.78 | Divorced | Some College or AA degree | Obesity | 4 | No | Yes | NO | 1 | 38 | Yes |
| 5459 | 6019 | >50 | Non-Hispanic White | 1.6 | Married | Less Than 9th Grade | Normal | 3 | No | Yes | NO | 1 | 46 | Yes |
| 6418 | 7077 | >50 | Non-Hispanic White | 1.78 | Married | High School Grad/GED or Equivalent | Obesity | 5 | Yes | No | NO | 1 | 63 | Yes |
| 6680 | 7377 | >50 | Mexican American | 1.37 | Married | Less Than 9th Grade | Obesity | 4 | Yes | Yes | NO | 1 | 58 | Yes |
| 8575 | 9467 | >50 | Non-Hispanic White | 1.01 | Married | 9-11th Grade (Includes 12th grade with no diploma) | Normal | 2 | No | Yes | NO | 1 | 78 | Yes |
| 8663 | 9566 | >50 | Non-Hispanic White | 2.58 | Married | Less Than 9th Grade | Underweight | 4 | No | Yes | NO | 1 | 55 | Yes |
| 10455 | 11572 | >50 | Mexican American | 1.64 | Married | Less Than 9th Grade | Normal | 3 | Yes | No | NO | 1 | 46 | Yes |
| 10491 | 11608 | >50 | Non-Hispanic Black | 0.58 | Divorced | 9-11th Grade (Includes 12th grade with no diploma) | Normal | 9 | Yes | Yes | NO | 1 | 47 | Yes |
| 11099 | 12294 | >50 | Other Race - Including Multi-Racial | 5 | Divorced | High School Grad/GED or Equivalent | Normal | 2 | Yes | Yes | NO | 1 | 52 | Yes |
| 11487 | 12733 | >50 | Other Hispanic | 0.34 | Never married | 9-11th Grade (Includes 12th grade with no diploma) | Normal | 4 | No | No | NO | 1 | 53 | No |
| 11622 | 12890 | >50 | Non-Hispanic White | 0.6 | Divorced | 9-11th Grade (Includes 12th grade with no diploma) | Normal | 7 | Yes | Yes | NO | 1 | 50 | Yes |
| 14256 | 15865 | <=50 | Non-Hispanic White | 5 | Never married | High School Grad/GED or Equivalent | Normal | 1 | Yes | Yes | NO | 1 | 50 | Yes |
| 15840 | 17644 | >50 | Non-Hispanic White | 0.93 | Married | Less Than 9th Grade | Underweight | 5 | No | No | NO | 1 | 78 | Yes |
| 17228 | 19199 | >50 | Non-Hispanic White | 3.32 | Married | Some College or AA degree | Obesity | 4 | Yes | Yes | NO | 1 | 25 | Yes |
| 17444 | 19441 | >50 | Non-Hispanic White | 2.37 | Divorced | Some College or AA degree | Obesity | 8 | Yes | Yes | NO | 1 | 64 | Yes |
| 17843 | 19896 | >50 | Non-Hispanic White | 2.33 | Divorced | High School Grad/GED or Equivalent | Normal | 4 | Yes | Yes | NO | 1 | 76 | No |
| 19109 | 21309 | >50 | Non-Hispanic White | 0.06 | Divorced | 9-11th Grade (Includes 12th grade with no diploma) | Normal | 4 | No | Yes | NO | 1 | 60 | Yes |
| 20512 | 22867 | >50 | Non-Hispanic White | 1.44 | Married | 9-11th Grade (Includes 12th grade with no diploma) | Normal | 5 | No | No | Yes | 1 | 31 | No |
| 22718 | 25325 | >50 | Non-Hispanic White | 1.94 | Divorced | 9-11th Grade (Includes 12th grade with no diploma) | Normal | 4 | No | Yes | NO | 1 | 60 | Yes |
| 22987 | 25627 | >50 | Non-Hispanic White | 2.58 | Divorced | High School Grad/GED or Equivalent | Obesity | 2 | Yes | Yes | Yes | 1 | 85 | Yes |
| 25338 | 28253 | >50 | Non-Hispanic White | 1.67 | Divorced | 9-11th Grade (Includes 12th grade with no diploma) | Normal | 2 | Yes | Yes | NO | 1 | 83 | No |
| 25372 | 28291 | >50 | Non-Hispanic White | 1.12 | Married | Less Than 9th Grade | Normal | 8 | No | Yes | NO | 1 | 50 | Yes |
| 27481 | 30592 | <=50 | Non-Hispanic White | 0.96 | Married | High School Grad/GED or Equivalent | Obesity | 3 | No | No | NO | 1 | 44 | No |
| 30172 | 33604 | >50 | Non-Hispanic White | 2.27 | Married | 9-11th Grade (Includes 12th grade with no diploma) | Normal | 6 | Yes | Yes | NO | 1 | 49 | Yes |
| 31385 | 34960 | >50 | Non-Hispanic White | 0.76 | Divorced | High School Grad/GED or Equivalent | Obesity | 9 | Yes | Yes | NO | 1 | 99999 | Yes |
| 33780 | 37618 | >50 | Non-Hispanic Black | 1.67 | Married | Some College or AA degree | Obesity | 4 | No | Yes | NO | 1 | 53 | Yes |
| 33837 | 37683 | <=50 | Non-Hispanic Black | 0.84 | Divorced | High School Grad/GED or Equivalent | Obesity | 2 | No | No | NO | 1 | 44 | No |
| 35069 | 39060 | >50 | Non-Hispanic White | 1.56 | Married | 9-11th Grade (Includes 12th grade with no diploma) | Underweight | 4 | No | Yes | NO | 1 | 72 | Yes |
| 39030 | 43441 | >50 | Non-Hispanic White | 1.64 | Married | 9-11th Grade (Includes 12th grade with no diploma) | Obesity | 4 | No | No | Yes | 1 | 45 | Yes |
| 39104 | 43524 | >50 | Non-Hispanic Black | 2.29 | Divorced | Less Than 9th Grade | Normal | 7 | Yes | No | NO | 1 | 72 | Yes |
| 39457 | 43916 | >50 | Non-Hispanic White | 2.14 | Married | 9-11th Grade (Includes 12th grade with no diploma) | Normal | 1 | No | No | NO | 1 | 45 | Yes |
| 40377 | 44920 | >50 | Non-Hispanic Black | 0.54 | Divorced | High School Grad/GED or Equivalent | Normal | 5 | Yes | Yes | NO | 1 | 73 | Yes |
| 40979 | 45577 | <=50 | Non-Hispanic Black | 0.34 | Divorced | 9-11th Grade (Includes 12th grade with no diploma) | Normal | 3 | Yes | No | NO | 1 | 20 | Yes |
| 41924 | 46634 | >50 | Non-Hispanic White | 1.25 | Divorced | Some College or AA degree | Normal | 4 | Yes | Yes | NO | 1 | 53 | Yes |
| 42183 | 46922 | >50 | Other Hispanic | 1.31 | Divorced | Less Than 9th Grade | Normal | 2 | Yes | Yes | NO | 1 | 48 | Yes |
| 42537 | 47308 | <=50 | Non-Hispanic Black | 0.76 | Never married | 9-11th Grade (Includes 12th grade with no diploma) | Normal | 5 | Yes | Yes | NO | 1 | 55 | Yes |
| 43675 | 48576 | >50 | Non-Hispanic White | 0.86 | Married | High School Grad/GED or Equivalent | Obesity | 6 | No | Yes | Yes | 1 | 57 | Yes |
| 45007 | 50031 | >50 | Other Hispanic | 0.94 | Divorced | Less Than 9th Grade | Obesity | 5 | No | No | NO | 1 | 68 | No |
| 45645 | 50730 | >50 | Other Race - Including Multi-Racial | 0.77 | Divorced | Less Than 9th Grade | Underweight | 1 | Yes | Yes | Yes | 1 | 20 | Yes |
| 49029 | 54392 | <=50 | Non-Hispanic Black | 5 | Never married | Some College or AA degree | Obesity | 4 | Yes | Yes | NO | 1 | 55 | Yes |
| 49096 | 54467 | >50 | Mexican American | 0.21 | Divorced | Less Than 9th Grade | Normal | 10 | Yes | Yes | NO | 1 | 65 | Yes |
| 49628 | 55047 | >50 | Non-Hispanic White | 5 | Married | Some College or AA degree | Obesity | 1 | Yes | Yes | Yes | 1 | 58 | Yes |
| 50334 | 55822 | >50 | Non-Hispanic White | 1.09 | Married | 9-11th Grade (Includes 12th grade with no diploma) | Normal | 2 | Yes | Yes | NO | 1 | 44 | Yes |
| 51412 | 56989 | <=50 | Non-Hispanic White | 0.81 | Married | 9-11th Grade (Includes 12th grade with no diploma) | Obesity | 5 | No | No | NO | 1 | 28 | Yes |
| 54096 | 59943 | >50 | Non-Hispanic White | 1.3 | Divorced | Some College or AA degree | Normal | 6 | Yes | Yes | NO | 1 | 65 | Yes |
| 54202 | 60057 | >50 | Other Race - Including Multi-Racial | 1.69 | Married | Some College or AA degree | Obesity | 5 | Yes | Yes | NO | 1 | 57 | Yes |
| 55251 | 61207 | >50 | Non-Hispanic White | 0.75 | Divorced | 9-11th Grade (Includes 12th grade with no diploma) | Obesity | 3 | Yes | No | NO | 1 | 54 | Yes |
| 56719 | 62821 | >50 | Non-Hispanic Black | 1.88 | Divorced | High School Grad/GED or Equivalent | Obesity | 3 | Yes | No | NO | 1 | 46 | Yes |
| 57149 | 63285 | >50 | Non-Hispanic White | 1.16 | Divorced | High School Grad/GED or Equivalent | Normal | 5 | Yes | No | NO | 1 | 71 | Yes |
| 58901 | 65190 | >50 | Non-Hispanic White | 1.78 | Divorced | High School Grad/GED or Equivalent | Normal | 3 | Yes | Yes | NO | 1 | 47 | Yes |
| 62088 | 68645 | >50 | Non-Hispanic White | 2.51 | Divorced | College Graduate or above | Normal | 3 | Yes | No | Yes | 1 | 50 | No |
| 64006 | 70733 | >50 | Non-Hispanic Black | 0.92 | Divorced | Less Than 9th Grade | Normal | 2 | Yes | Yes | NO | 1 | 40 | Yes |
| 65732 | 74262 | >50 | Non-Hispanic White | 0.74 | Divorced | High School Grad/GED or Equivalent | Normal | 5 | Yes | No | NO | 1 | 60 | Yes |
| 66086 | 74668 | >50 | Non-Hispanic White | 1.71 | Divorced | 9-11th Grade (Includes 12th grade with no diploma) | Normal | 2 | No | No | NO | 1 | 14 | Yes |
| 68282 | 77067 | >50 | Non-Hispanic White | 3.03 | Married | College Graduate or above | Normal | 1 | Yes | Yes | NO | 1 | 61 | Yes |
| 68715 | 77533 | >50 | Non-Hispanic White | 0.99 | Divorced | 9-11th Grade (Includes 12th grade with no diploma) | Normal | 4 | Yes | Yes | NO | 1 | 35 | Yes |
| 69045 | 77896 | <=50 | Non-Hispanic White | 0.25 | Divorced | 9-11th Grade (Includes 12th grade with no diploma) | Normal | 5 | Yes | No | NO | 1 | 35 | Yes |
| 69587 | 78484 | >50 | Non-Hispanic White | 0.64 | Divorced | Some College or AA degree | Normal | 3 | No | No | NO | 1 | 55 | Yes |
| 69670 | 78580 | >50 | Non-Hispanic White | 1.04 | Divorced | 9-11th Grade (Includes 12th grade with no diploma) | Normal | 7 | Yes | Yes | NO | 1 | 75 | Yes |
| 71182 | 80222 | >50 | Non-Hispanic White | 0.82 | Married | 9-11th Grade (Includes 12th grade with no diploma) | Normal | 3 | Yes | No | NO | 1 | 50 | Yes |
| 73426 | 82655 | <=50 | Other Race - Including Multi-Racial | 2.32 | Married | Less Than 9th Grade | Obesity | 11 | Yes | No | NO | 1 | 75 | No |
| 75553 | 85003 | <=50 | Mexican American | 1.35 | Married | Less Than 9th Grade | Obesity | 3 | No | Yes | NO | 1 | 57 | No |
| 76785 | 86358 | >50 | Non-Hispanic White | 2.1 | Married | High School Grad/GED or Equivalent | Obesity | 2 | Yes | Yes | NO | 1 | 49 | Yes |
| 77862 | 87545 | >50 | Other Race - Including Multi-Racial | 0.94 | Divorced | Some College or AA degree | Normal | 5 | Yes | Yes | Yes | 1 | 38 | Yes |
| 78825 | 88593 | >50 | Non-Hispanic White | 5 | Divorced | Some College or AA degree | Normal | 5 | Yes | Yes | NO | 1 | 55 | Yes |
| 79235 | 89052 | >50 | Other Hispanic | 0.57 | Married | Some College or AA degree | Obesity | 2 | Yes | No | NO | 1 | 72 | Yes |
| 80166 | 90068 | >50 | Other Hispanic | 0.57 | Married | Some College or AA degree | Obesity | 1 | No | No | NO | 1 | 68 | Yes |
| 80709 | 90653 | >50 | Other Hispanic | 2.82 | Married | Some College or AA degree | Normal | 2 | No | No | NO | 1 | 56 | Yes |
