# Supplementary material for: Association Between Emphysema and Breast Cancer: Data from National Health and Nutrition Examination Survey (1998–2016)
Source: Womens Health Rep (New Rochelle). 2025 Jul 15;6(1):681–90. doi: 10.1177/26884844251359511 (PMC12479188; doi:10.1177/26884844251359511)
Supplement: Supplementary Table S3 [file 26884844251359511_supplementary_table_s3.docx]

Supplementary Table 3: Baseline statistical table

| Characteristics | state | normal | emphysema | pvalue |
| --- | --- | --- | --- | --- |
| COPD | No | 16245 (97.4) | 97 (29.8) | <0.001 |
|  | Yes | 440 (2.6) | 228 (70.2) | <0.001 |
| Stomach cancer | No | 3 (21.4) | 0 (0) | 1.000 |
|  | Yes | 11 (78.6) | 1 (100) | 1.000 |
| Melanoma | No | 3 (2.4) | 0 (0) | 1.000 |
|  | Yes | 122 (97.6) | 3 (100) | 1.000 |
| Colon cancer | No | 2 (1.8) | 0 (0) | 1.000 |
|  | Yes | 110 (98.2) | 5 (100) | 1.000 |
| Uterus cancer | No | 3 (4.2) | 0 (0) | 1.000 |
|  | Yes | 68 (95.8) | 6 (100) | 1.000 |
| Skin cancer | No | 3 (1.2) | 0 (0) | 1.000 |
|  | Yes | 250 (98.8) | 9 (100) | 1.000 |
| lung cancer | No | 2 (4.8) | 0 (0) | 0.608 |
|  | Yes | 40 (95.2) | 15 (100) | 0.608 |
