# Supplementary material for: Association Between Emphysema and Breast Cancer: Data from National Health and Nutrition Examination Survey (1998–2016)
Source: Womens Health Rep (New Rochelle). 2025 Jul 15;6(1):681–90. doi: 10.1177/26884844251359511 (PMC12479188; doi:10.1177/26884844251359511)
Supplement: Supplementary Table S4 [file 26884844251359511_supplementary_table_s4.docx]

Supplementary Table 4: Distribution of liver cancer samples

| Emphysema | Liver_cancer |
| --- | --- |
| No | Yes |
| No | Yes |
| No | No |
| No | No |
| No | Yes |
| No | Yes |
| No | Yes |
| No | Yes |
| No | Yes |
| No | Yes |
| No | Yes |
| No | Yes |
| No | Yes |
| No | Yes |
| No | Yes |
| No | Yes |
| No | No |
